# Description of thyroid disorders the year before conception: a population-based study

**DOI:** 10.3389/fendo.2023.1236505

**Published:** 2023-09-25

**Authors:** Glòria Tena Vivó, Neus Parellada Esquius, Oriol Cunillera Puértolas, Mercè Albareda Riera, Mónica Isidro Albaladejo, Lluís Vila Ballester

**Affiliations:** ^1^ Hospital de Viladecans, Obstetrics & Gynecology Viladecans, Barcelona, Spain; ^2^ Institut Català de la Salut Gerència Territorial Metropolitana Sud, Epidemiology and Research, L’Hospitalet de Llobregat, Catalunya, Spain; ^3^ Unitat de Suport a la Recerca Metropolitana Sud, Fundació Institut Universitari per a la recerca a l’Atenció Primària de Salut Jordi Gol i Gurina, l’Hospitalet de Llobregat, Catalunya, Spain; ^4^ Endocrinology and Nutrition Division, Hospital de Sant Joan Despí Moisès Broggi, Sant Joan Despí, Spain; ^5^ Institut Catalá de la Salut (ICS), Sexual and Reproductive Primary Health Care, Sant Boi de Llobregat, Spain

**Keywords:** thyroid disorders, preconception, prevalence, hyperthyroidism, hypothyroidism

## Abstract

**Objective:**

This study aimed to monitoring the prevalence of previously identified thyroid disorders and hypothyroidism monitoring before pregnancy.

**Material and methods:**

A retrospective cross-sectional study of women whose pregnancies occurred between 2014 and 2016 was conducted, including 120,763 pregnancies in Catalonia (Spain). The presence of thyroid disorders in women was based on disease diagnostic codes and/or prescription of levothyroxine or antithyroid drugs. To evaluate the thyroid disorder diagnosis and monitoring, thyrotropin (TSH), free T4 (FT4), antiperoxidase antibody (TPOAb), and anti-TSH receptor antibody (TRAb) records were gathered and categorised according to the reference values of each laboratory.

**Results:**

The prevalence of recorded thyroid disorders before the last menstrual period was 5.09% for hypothyroidism and 0.64% for hyperthyroidism,showing a significant increase with age. A thyroid monitoring test was not performed in the year before the last menstrual period in approximately 40% of women with a known thyroid disorder. Amongst the women with hypothyroidism who underwent a TSH test, 31.75% showed an above-normal result. Amongst women previously unknown to have thyroid disorders, 3.12% had elevated TSH levels and 0.73% had low TSH levels.

**Conclusion:**

A high percentage of Catalan women with a known thyroid disorder were not properly monitored during the year before pregnancy. Amongst those monitored, more than one-third had TSH values outside the reference range. Therefore, it is important to evaluate women with thyroid disorders during pre-pregnancy visits.

## Introduction

Thyroid disorders (TDs) are a global health problem that affect patients, depending on the type and severity of the disorder. They can have significant adverse effects on pregnant women and their offspring.

Numerous worldwide studies have shown different epidemiological TD data depending on the illness definition, methods used to measure thyroid hormones, reference ranges applied, and target population (1, 2).

Nonetheless, the prevalence of hyperthyroidism in the general population is similar to that in the USA and Europe, at approximately 0.6% for overt hyperthyroidism (3) and 1% to 5% for subclinical hyperthyroidism. The prevalence of hypothyroidism ranges from 0.2% to 5.3% in Europe and from 0.3% to 3.7% in the USA (2, 4). A Spanish study (1) reported a 0.9% prevalence of hyperthyroidism and a 9.1% prevalence of hypothyroidism. Both hyperthyroidism and hypothyroidism are prevalent in women (1, 3–6).

TD is the second most common endocrinopathy during pregnancy (7). Adequate monitoring and treatment can be consequential for pregnancy and foetus outcomes.

For TD patients, clinical guidelines (8–10) recommend monitoring the thyroid function prior to pregnancy. However, few studies have been conducted to determine the implementation of this recommendation (7, 11, 12). In Catalonia, the TD prevalence amongst young women, the monitoring rate before pregnancy in women with existing TD conditions, and clinical guideline compliance are unknown.

Hypothyroidism is generally monitored and treated during primary care settings. Hyperthyroidism cases are controlled by specialised care in Catalonia.

This study aimed to evaluate the prevalence of previously identified TD and hypothyroidism monitoring before pregnancy in women who became pregnant in Catalonia.

## Materials and methods

This population-based cross-sectional study of pregnancies based on secondary data was performed. The data were obtained from computerized records of the Information System for Research Development in Primary Care (www.sidiap.org). SIDIAP generates a comprehensive amount of medical data and provides access to valid research information. The SIDIAP includes diagnostic and prescription data from both primary care and hospitals (except for propyl-thyouracyl, which is only dispensed in hospitals). Laboratory data are from primary care only, and not from specialised or private centres.

Jordi Gol (P17/113) Clinical Research Ethical Committee approved the protocol. Confidentiality was ensured since all the cases in the SIDIAP database are pseudo-anonymous, thus the informed consent from participants was not required.

According to previous studies, the prevalence of TD identified prior to pregnancy is 8.9% in the population aged 18–75 years (5). There are no studies in Catalonia based exclusively on young women. Thus, to attain the main aim of this study, with an alpha risk factor of 5% and a precision of 2%, a minimum of 779 pregnant women are necessary.

According to the Statistical Institute of Catalonia (IDESCAT), 210,943 childbirths were registered in 2014–2016 [2014-2016.Institut d’Estadística de Catalunya. IDESCAT. http://www.idescat.cat/pub/?id=aec&n=259&t=2015”\h].

In the present study, all clinical records from the SIDIAP database were analysed. The SIDIAP database included 80% of the Catalan population. The number of cases studied exceeded the minimum required sample size.

During 2014–2016, gestations of women who attended primary health care were studied. Women younger than 16 years or older than 49 years of age were excluded.

The presence of TD in women before pregnancy was based on disease diagnostic codes, but also considering the prescription of thyroid hormones or antithyroid drugs the year before the last menstrual period (LMP). The LMP was calculated on the first trimester ultrasound.

Hypothyroidism before pregnancy was confirmed if, at the LMP, there was an active prescription of thyroid hormones (ATC H03AA code-levothyroxine) or any of the following diagnoses (CIE-10): E00, congenital iodine-deficiency syndrome; E02, subclinical iodine-deficiency hypothyroidism; E03, other hypothyroidism; E89.0, post procedural hypothyroidism (total thyroidectomy and post I-131 ablation).

Additionally, hyperthyroidism prior to pregnancy was confirmed if there was an active prescription of antithyroid drugs (ATC H03B code) or an E05 Thyrotoxicosis code at the LMP.

Other TD diagnostics were also collected: E04 other nontoxic goitre, E07 other disorders of the thyroid, E06 thyroiditis, E01 iodine-deficiency related thyroid disorders and allied conditions, and C73 malignant neoplasm of the thyroid gland.

Participants were classified according to their thyroid disorders as hypothyroidism, hyperthyroidism, other thyroid disorders, or unknown thyroid disorders.

When multiple diagnoses or prescriptions for TD were registered, only the last active diagnosis was considered for the classification.

To evaluate the diagnosis and monitoring of TD, thyrotropin (TSH), free T4 (FT4), antiperoxidase antibodies (TPOAb), and anti-TSH receptor antibody (TRAb) records were collected the year before conception. In the case of several controls, only the one closer to the LMP was considered. A total of 15 different laboratory techniques were used, mainly enzymechemiluminescence, electrochemiluminescence, and immuno-chemiluminescence. General population reference values (GPRV) for TSH ranged between 0.2 mIU/L–4.2 mIU/L and 0.34 mIU/L–5.6 mIU/L, and for FT4 they ranged between 0.54 ng/dL–1.24 ng/dL and 0.78 ng/dL–2.02 ng/dL.

TSH and FT4 analytical results were categorized as below normal, within the normal range, or above normal. TPOAb and TRAb results were considered positive if the value was higher than the upper limit of the GPRV.

Overt hyperthyroidism was defined as low TSH with an elevated FT4. Subclinical hyperthyroidism was defined as low TSH and normal FT4 levels. Overt hypothyroidism was defined as an elevated TSH with low FT4. Subclinical hypothyroidism was defined as elevated TSH with normal FT4 levels, using GPRV.

Following ATA 2011 guidelines, a TSH target <2.5 mIU/L was defined for women with treated hypothyroidism and pregnancy desire.

The age at LMP was determined for all participants, as well as the presence of comorbidities: hypertension (I10 and I15), diabetes (E11, E12, E13, E14), obesity (BMI = o > 30 Kg/m^2^) (E66), morbid obesity (BMI = o > 40 Kg/m^2^) (E66.8), and ischaemic heart disease (I20, I21, I22, I23, I24, I25).

Descriptive statistical analysis was conducted with medians and interquartile ranges (IQR) for quantitative variables and absolute and relative frequencies for qualitative variables.

Bivariate statistics were used to evaluate differences amongst TD types, and the chi-square test or variance analysis was used depending on the scale variables.

The R-4.0.3 statistical package was used for this analysis.

## Results

From 2014 to 2016, 134,860 pregnancies were registered in the SIDIAP database. Having applied the exclusion criteria, 120,763 pregnancies were included. The participants’ mean age was 31 (IQR 27.00-35.00).

**Figure 1:**
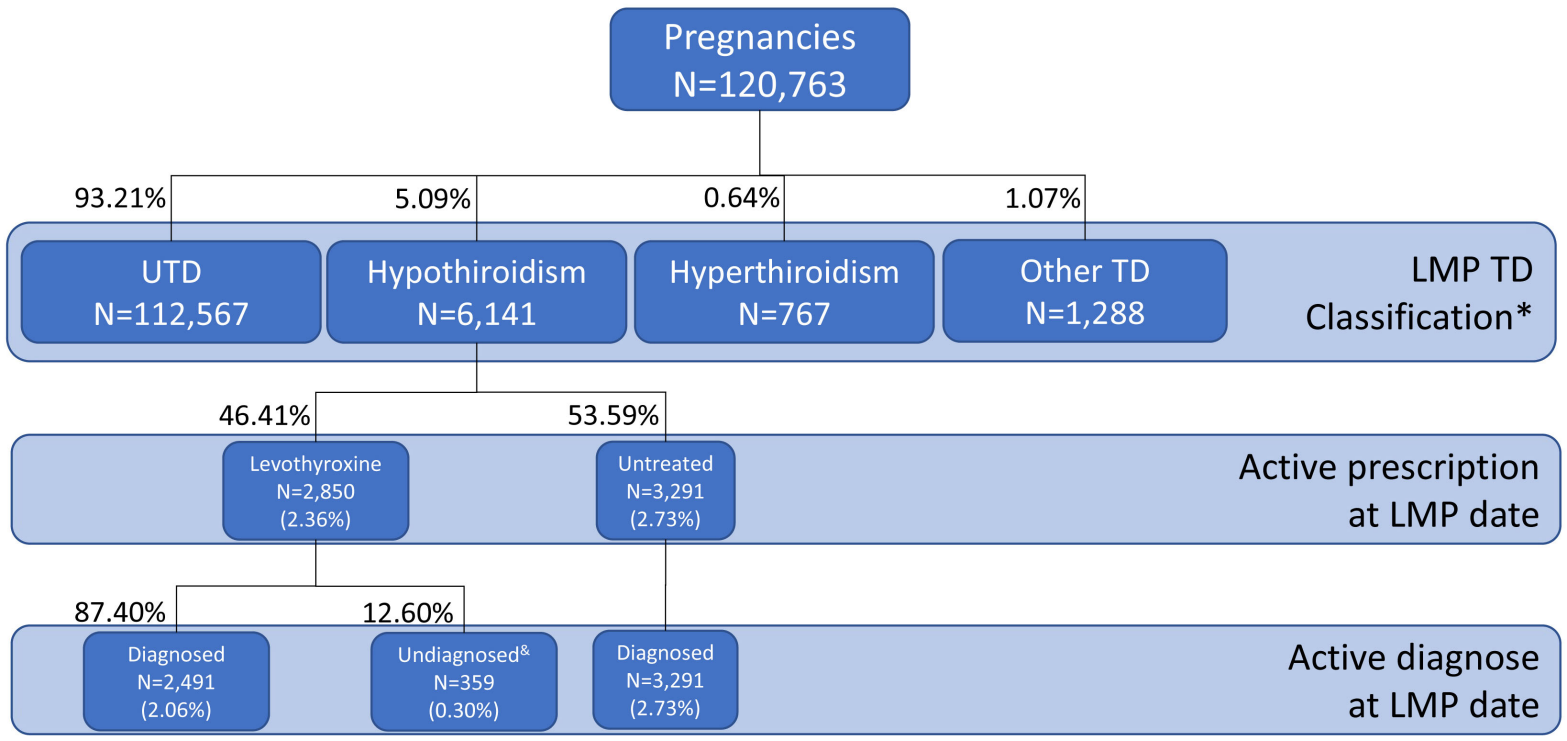
Distribution of thyroid dysfunction based on registered diagnostic codes and active treatment. LMP, Last Menstrual Period; TD, thyroid dysfunction; UTD, Unknown TD. *Based on last diagnostic or treatment evidence prior to –and active at- LMP date; no lab test results considered.^&^Patients in treatment not included on the registry of active diagnoses.

The total TD prevalence in the year before conception was 6.80%: 5.09% for hypothyroidism, 0.64% for hyperthyroidism, and 1.07% for other TD (**Figure 1**).

Patients with hypothyroidism were prescribed levothyroxine in 46.41% of the cases; thus, the treated hypothyroidism prevalence was estimated to be 2.36%. Levothyroxine was prescribed with no related diagnostic code in 0.30% of the participants (n = 359) (**Figure 1**).

Hypothyroidism prevalence was higher amongst women with diabetes, hypertension, or obesity (**Table 1**). The prevalence of hyperthyroidism was also higher in participants with diabetes. TD prevalence increased with age (**Table 1**).

**Table 1:** Prevalence of known thyroid disorders and associated morbidity at the date of the last menstrual period (TD, thyroid disorder).

	Global (n = 120,763)	Unknown TD (n = 112,567)	Hypothyroidism (n = 6,141)	Hyperthyroidism (n = 767)	Other thyroid disorders (n = 1,288)
Age (years)					
*[16, 18]*	1,012 (0.84%)	989 (97.73%)	19 (1.88%)	4 (0.40%)	0 (0.00%)
*[18, 30]*	44,215 (36.61%)	42,001 (94.99%)	1,705 (3.86%)	200 (0.45%)	309 (0.70%)
*[30, 40]*	67,905 (56.23%)	62,722 (92.37%)	3,829 (5.64%)	497 (0.73%)	857 (1.26%)
*[40, 49]*	7,631 (6.32%)	6,855 (89.84%)	588 (7.71%)	66 (0.86%))	122 (1.60%)
**Total**	**120,763 (100%)**	**112,567 (93.21%)**	**6,141 (5.09%)**	**767 (0.64%)**	**1,288 (1.07%)**
**Ischaemic heart disease**	18 (0.01%)	15 (83.33%)	1 (5.56%)	2 (11.11%)	0 (0.00%)
**Diabetes Mellitus**	564 (0.47%)	458 (81.21%)	87 (15.43%)	13 (2.30%)	6 (1.06%)
**Hypertension**	1,038 (0.86%)	910 (87.67%)	104 (10.02%)	9 (0.87%)	15 (1.45%)
**Obesity**	10,663 (8.83%)	9,532 (89.39%)	907 (8.51%)	83 (0.78%)	141 (1.32%)
**Morbid Obesity**	383 (0.32%)	329 (85.90%)	47 (12.27%)	1 (0.26%)	6 (1.57%)

### Primary care thyroid function records

During the year before conception, TSH measurement was requested for 64.16% of women with hypothyroidism (**Table 2**); this figure reached 76.9% in patients with hypothyroidism who were on levothyroxine treatment (**Table 3**). TSH was recorded in 20.76% of participants with previously unknown thyroid disorders (UTD); this group had a greater prevalence of diabetes (0.84% vs 0.29%), hypertension (1.29% vs 0.68%), and obesity (11.57% vs 7.89%) prevalence.

**Table 2:** Request for tests to monitor thyroid function and autoimmunity in the year prior to Last Menstrual Period date.

Test Required	UTD (n = 112,567)	Hypothyroidism (n = 6,141)	Other thyroid disorders (n = 1,288)
**FT4**	5,195 (4.62%)	3,223 (52.48%)	316 (24.53%)
**TSH**	23,366 (20.76%)	3,940 (64.16%)	580 (45.03%)
**acTPO**	331 (0.29%)	481 (7.83%)	64 (4.97%)
**TRAb**	14 (0.01%)	30 (0.49%)	10 (0.78%)

TD, Thyroid Disorder; UTD, unknown TD; FT4, Free Thyroxin; TSH, Thyrotropin; acTPO, antiperoxidase antibodies; TRAb, anti-TSH receptor antibody.

**Table 3:** Differences in baseline laboratory testing between treated and non-treated patients with hypothyroidism.

Test	Without prescription (n = 3,291)	With prescription (n = 2,850)	p-value
**FT4**	1,305 (39.65%)	1,918 (67.30%)	<0.001
**TSH**	1,748 (53.11%)	2,192 (76.91%)	<0.001
**acTPO**	186 (5.65%)	295 (10.35%)	<0.001
**TRAb**	11 (0.33%)	19 (0.67%)	0.068

FT4, Free Thyroxin; TSH, Thyrotropin; acTPO, antiperoxidase antibodies; TRAb, anti-TSH receptor antibody.

### Thyroid function monitoring

#### Participants with known hypothyroidism

**Figure 2:**
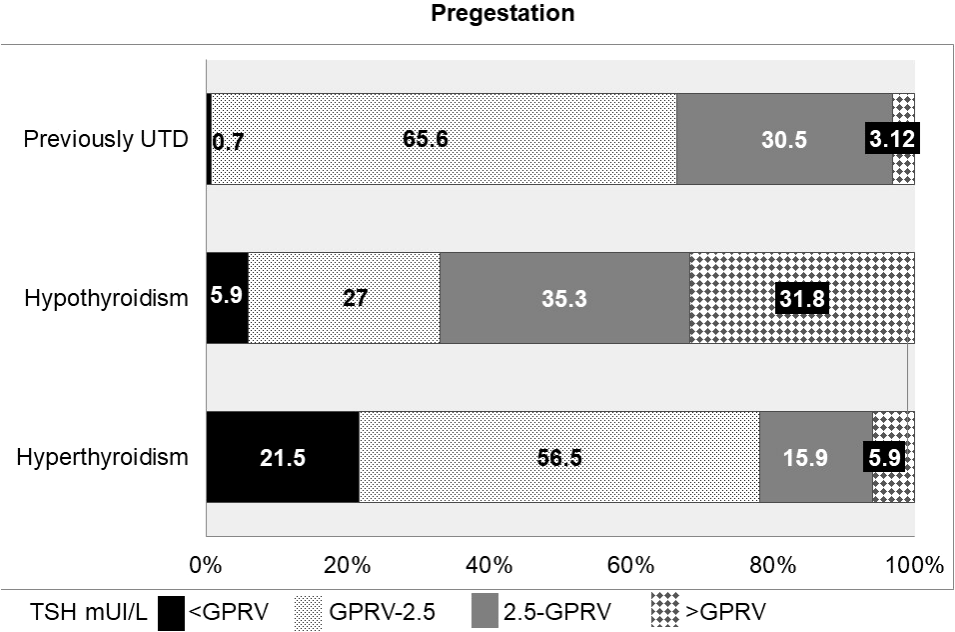
TSH distribution in the population with known Thyroid Disorder. UTD, Unknown Thyroid Disorder; GPRV, General Population Reference Values.

From 6,141 women with known hypothyroidism, 3,940 (64.16%) had a TSH level recorded in the year before conception. The TSH level was above the GPRV in 31.75% of the patients. Amongst women with hypothyroid treated with thyroxine, 42.34% had a TSH <2.5 mUI/L. The FT4 was analysed in 3,208 women and it was found to be low in 2.8% of them. Amongst the treated participants with TSH measurements, 9% had a low TSH level (7.25% with normal FT4, 1.64% with high FT4 and 0.14% without FT4 measurement). **Figure 2** shows the TSH distribution in the population with hypothyroidism and unknown TD.

#### Other thyroid disorders

Amongst the 1,288 patients with other thyroid disorders, TSH was analysed in 543 cases (42.1%). Of these, 3.97% showed high TSH levels and 2.14% showed low TSH levels. In 313 women, FT4 was also analysed. This group displayed 0.95% (3/313) of overt hypothyroidism and 0.64% (2/313) of overt hyperthyroidism.

#### Participants with unknown thyroid disorders (UTD)

TSH was assessed during the year before conception in 23,366 women (20.76%) with UTD. In this group, 0.73% had low TSH levels and 3.12% had high TSH levels. Of the total, 5,181 also had FT4 measurements, resulting in 0.05% displaying overt hyperthyroidism and 2.23% subclinical hyperthyroidism. The same group also displayed overt (0.63%) and subclinical (9.51%) hypothyroidism.

#### Autoimmunity

Positive TPOAb prevalence amongst UTD participants was 16.92% (56/331), and amongst women with hypothyroidism, hyperthyroidism and remaining disorders groups, 52.39% (252/481), 47.83% (33/69), and 32.81% (21/64), respectively.

### Thyroid dysfunction prevalence

The prevalence of known and unknown hypothyroidism was 5.09% and 3.12%, respectively, whereas that of hyperthyroidism the corresponding figures was 0.64% and 0.73%, respectively. Therefore, the total thyroid dysfunction prevalence in childbearing age women who became pregnant was 9.58% in Catalonia.

## Discussion

This study analyses the prevalence of TD prior to pregnancy and hypothyroidism monitoring in the year before conception in Catalonia (Spain). The total prevalence of thyroid dysfunction in these individuals was 9.58%. Almost 40% of participants with known hypothyroidism did not have thyroid function monitored the year before conception. In addition, TSH measurements were outside the desirable preconception target range in one-third of participants with hypothyroidism.

The prevalence of TD in the UTD group was 3.9%. Knowing the adverse outcomes of uncontrolled thyroid disorders in pregnancy, this percentage could lead to the consideration of universal screening for thyroid disorders in pregnant women.

The prevalence of known TD (diagnosed and/or treated) in this area was 5.09% and 0.64% for hypothyroidism and hyperthyroidism, respectively. The prevalence of thyroid dysfunction is similar to that reported by Leese et al. [11] for hypothyroidism (5.14%) but lower for hyperthyroidism (1.26%). The Tayside cohort [6, 12] showed a higher prevalence of hypothyroidism (9.3%) and hyperthyroidism (3.9%), in women than in the present study. However, only 13.2% of the Tayside’s women were under 45 years of age. Hence, the older age and primary data used by Leese might explain these differences.

Torrejón et al. [13], report a prevalence of known hypothyroidism, in women aged 30–50 of 3.6%. If we disregard the patients treated without a diagnosis of hypothyroidism, as Torrejón et al. did, the percentage of hypothyroidism is 4.8%, which is higher. In the case of hyperthyroidism, the results obtained in this study are similar to the range identified by Torrejón for women aged between 30 and 50 years old (0.54%) (13).

The observed hypothyroidism prevalence (5.09%) was similar to the data published for the European population by Taylor et al. (0.2%–5.3%) (2). However, it is significantly lower than that reported by Valdés et al. (9.1%) (1). The differences may be due to the different population age ranges as well as the methodology used, since Valdés uses primary data.

Regarding the UTD group, the results of overt hypothyroidism were similar to those of a Spanish study (1) (0.6% vs. 0.64%). In contrast, a different Catalan study (5) detected a higher prevalence of hypothyroidism (3.8% vs. 3.12%) and hyperthyroidism (1.5% vs. 0.73%) in women. This could be due to the primary data used, which generally detects more cases than those present in the diagnostic records.

The epidemiology of thyroid disorders depends on multiple factors such as nutritional iodine intake and thyroid autoimmunity. However, the Catalan population fulfils the iodine intake of WHO recommended standards (1, 5, 13–15). Although the TPOAb prevalence detected was relatively high in the UTD group (16.92%), it could be associated with an adequate level of iodine status in the population (16), which is very similar to that obtained in Catalan women by Vila et al. (17.6%) (17).

It is worth noting that approximately 60% of the participants with known dysfunctions ran a TSH test in primary healthcare in the year before conception. This is especially alarming in the case of hypothyroidism, since it is mainly in Primary Healthcare, where this dysfunction is monitored in Catalonia. The exceptions are hypothyroidism due to thyroid cancer treatment or hypothyroidism with concomitant conditions, such as type 1 diabetes. These cases were followed up in the hospitals. However, they represented a low percentage of the total (1.1% thyroid cancer, 0.76% type 1 diabetes; data not shown). Hyperthyroidism cases were monitored in hospitals, and these data were not available in the database used.

Data on the follow-up preconception thyroid disorders are limited. A Canadian study analysed TSH levels 4 months prior to gestation in 10,680 women; amongst them, 44.4% had a TSH measurement (18). This is a lower figure than the one observed in the present study (64.16%), partly due to the shorter period considered (4 months vs. 12 months).

Therefore, the percentage of participants with known hypothyroidism who became pregnant without monitoring was clearly high (8–10). This can result in greater morbidity in both mothers and newborns (7, 19–27).

However, it is remarkable that the total number of tests requested for hypothyroidism control was significantly higher in participants on levothyroxine treatment. Hypothyroidism monitoring using TSH in these patients reached 76.91% (**Table 3**).

TSH analysis was prescribed to 20.76% of UTD participants during the year before conception. Although TSH measurement is recommended when diabetes is present (8, 9, 28), our results show that screening was performed in less than half of the participants with diabetes.

The percentage of participants with known hypothyroidism who had a high TSH level (31.75%) was very similar to that in a Canadian study (16), in which 30% of the women displayed TSH levels above their GPRV (4 mUI/L).

These figures highlight the poor compliance with the guidelines. International and local guidelines recommend monitoring yearly or more frequently if control is unsatisfactory (29, 30).

Many studies and guidelines support and recommend maintaining TSH <2.5 mUI/L in treated women with hypothyroidism prior to pregnancy (10, 31–33). However, in the present study, 57.66% of the treated hypothyroid women had a TSH level above 2.5 mUI/L the year before conception. In other studies, this figure was: Taylor et al. 46.1% (21), Kahn et al. 48.0% (31), and Lemieux et al. 52.4% (18).

The data we used did not provide information on whether the women included in the study were trying to become pregnant. However, this is not remarkable regarding the TSH measurement rate, since all people with TD should be screened at least once a year. Women of childbearing age with TD, regardless of whether they are seeking pregnancy, need to be followed-up regularly to achieve pregnancy with adequate TSH levels.

Additionally, our study shows that 5.9% of hypothyroid women display TSH levels below the GPRV. This is a high figure, albeit lower than the percentage obtained by Taylor et al. (23), where 13.6% of women were overtreated. Lemieix et al. (18) observed that overtreatment was associated with an increased risk of prematurity.

The results of this study were obtained from records not intended for research. The diagnostic codes are not always clear for clinical doctors. Furthermore, some codes may persist after successful treatment.

We do not know how many patients in the study were infertile and had become pregnant with assisted reproductive techniques. However, we do know that in Catalonia in 2014, 9,362 pregnancies were with assisted reproductive techniques out of 71,589 births (not pregnancies). From these, after excluding abortions and embryo reductions, 6,891 pregnancies could have been part of our study.

We did not have data on assisted reproductive techniques in Catalonia in 2015 and 2016 (34). This may be a limitation when discussing thyroxine treatment in women with hypothyroidism. LT4 therapy is often used in euthyroid patients if they are infertile with positive anti-TPO or have simple goitre, despite the lack of clear indications (10). However, given the small number of cases with these characteristics in relation to the total, the impact is low. The analysis of TSH and T4 cases assumes a bias, since T4 is usually requested automatically when TSH is over GPRV. This may lead to an overestimation of subclinical hypothyroidism.

In 2014, 25.4% of women aged 15–44 years in Catalonia had both public and private health coverage. We cannot know if any of the patients in the study were tested in the private health system

The strengths of this study are the high number of cases and the database used, which include more than 80% of the Catalan population (more than 7 million inhabitants).

## Conclusion

The known prevalence of hypothyroidism in the year before conception was 5.09%, which is in agreement with other studies.

Nearly 40% of Catalan women with TD who become pregnant do not undergo thyroid function testing the year before conception. In addition, TSH concentrations are outside the desirable preconception target range in one-third of the treated women with hypothyroidism.

It is imperative to make both childbearing-age Catalan women and their health professionals aware that preconception thyroid monitoring is unsatisfactory. They need to be conscious of the importance of a regular monitoring of TD (independent of whether women are intending pregnancy) to achieve gestation with adequate TSH levels.

The TD prevalence in the UTD women of childbearing age is 3.85%. Knowing the morbidity of uncontrolled TD during pregnancy could lead to the consideration of a universal screening for TD in pregnant women.

It can be concluded that there is a need to improve preconception monitoring of young women with known TD in Catalonia.

## Data availability statement

The data analyzed in this study is subject to the following licenses/restrictions: The data that support the findings of this study are not publicly available on legal or ethical grounds, as they contain information that could compromise the privacy of research participants, but are available from Oriol Cunillera Puértolas [O.C.P.] upon reasonable request. Requests to access these datasets should be directed to Oriol Cunillera Puértolas, ocunillera.idiap@gmail.com.

## Ethics statement

The studies involving humans were approved by Jordi Gol (P17/113) Clinical Research Ethical Committee approved the protocol. The studies were conducted in accordance with the local legislation and institutional requirements. The human samples used in this study were acquired from a by-product of routine care or industry. Written informed consent for participation was not required from the participants or the participants’ legal guardians/next of kin in accordance with the national legislation and institutional requirements.

## Author contributions

All the authors have contributed to the design of the work, revising it critically, have approved the last version, and agree to be accountable for the accuracy or integrity of any part of the work.
