# Supplementary material for: Description of thyroid disorders the year before conception: a population-based study
Source: Front Endocrinol (Lausanne). 2023 Sep 25;14:1236505. doi: 10.3389/fendo.2023.1236505 (PMC10561644; doi:10.3389/fendo.2023.1236505)
Supplement: Supplementary file 1 [file Table_1.docx]

Thyrotropin (TSH)

free T4 (FT4)

antiperoxidase antibodies (TPOAb)

anti-TSH receptor antibodies (TRAb)

last menstrual period date (LMP)

Thyroid disorders (TD)

General population reference values (GPRV)

interquartile ranges (IQR)

unknown thyroid disorders (UTD)
